# Thrombospondin-I is a critical modulator in non-alcoholic steatohepatitis (NASH)

**DOI:** 10.1371/journal.pone.0226854

**Published:** 2019-12-31

**Authors:** Jessica Min-DeBartolo, Franklin Schlerman, Sandeep Akare, Ju Wang, James McMahon, Yutian Zhan, Jameel Syed, Wen He, Baohong Zhang, Robert V. Martinez

**Affiliations:** 1 BioMedicine Design, Pfizer Worldwide Research and Development, Cambridge, Massachusetts, United States of America; 2 Department of Pharmacology & Experimental Therapeutics, Boston University School of Medicine, Boston, Massachusetts, United States of America; 3 Inflammation and Immunology Research Unit, Pfizer Worldwide Research and Development, Cambridge, Massachusetts, United States of America; 4 Drug Safety Research and Development, Pfizer Worldwide Research and Development, Groton, Connecticut, United States of America; 5 Early Clinical Development, Pfizer Worldwide Research and Development, Cambridge, Massachusetts, United States of America; Johns Hopkins University School of Medicine, UNITED STATES

## Abstract

Non-alcoholic fatty liver disease (NAFLD) is a progressive liver disease characterized by dysregulated lipid metabolism and chronic inflammation ultimately resulting in fibrosis. Untreated, NAFLD may progress to non-alcoholic steatohepatitis (NASH), cirrhosis and death. However, currently there are no FDA approved therapies that treat NAFLD/NASH. Thrombospondin-I (TSP-1) is a large glycoprotein in the extracellular matrix that regulates numerous cellular pathways including transforming growth factor beta 1 (TGF-β1) activation, angiogenesis, inflammation and cellular adhesion. Increased expression of TSP-1 has been reported in various liver diseases; however, its role in NAFLD/NASH is not well understood. We first examined TSP-1 modulation in hepatic stellate cell activation, a critical initiating step in hepatic fibrosis. Knockdown or inhibition of TSP-1 attenuated HSC activation measured by alpha smooth muscle actin (α-SMA) and Collagen I expression. To investigate the impact of TSP-1 modulation in context of NAFLD/NASH, we examined the effect of TSP-1 deficiency in the choline deficient L-amino acid defined high fat diet (CDAHFD) model of NASH in mice by assessing total body and liver weight, serum liver enzyme levels, serum lipid levels, liver steatosis, liver fibrosis and liver gene expression in wild type (WT) and TSP-1 null mice. CDAHFD fed mice, regardless of genotype, developed phenotypes of NASH, including significant increase in liver weight and liver enzymes, steatosis and fibrosis. However, in comparison to WT, CDAHFD-fed TSP-1 deficient mice were protected against numerous NASH phenotypes. TSP-1 null mice exhibited a decrease in serum lipid levels, inflammation markers and hepatic fibrosis. RNA-seq based transcriptomic profiles from the liver of CDAHFD fed mice determined that both WT and TSP-1 null mice exhibited similar gene expression signatures following CDAHFD, similar to biophysical and histological assessment comparison. Comparison of transcriptomic profiles based on genotype suggested that peroxisome proliferator activated receptor alpha (PPARα) pathway and amino acid metabolism pathways are differentially expressed in TSP-1 null mice. Activation of PPARα pathway was supported by observed decrease in serum lipid levels. Our findings provide important insights into the role of TSP-1 in context of NAFLD/NASH and TSP-1 may be a target of interest to develop anti-fibrotic therapeutics for NAFLD/NASH.

## Introduction

Liver fibrosis is a major cause of mortality worldwide [1]. It could also be a pathological manifestation of various disorders such as metabolic imbalance, viral or parasitic infection and exposure to toxins such as excess alcohol [2]. A prolonged state of inflammation and fibrosis without proper injury resolution in the liver may ultimately lead to cirrhosis, hepatic carcinoma and death. Regression of hepatic fibrosis has been observed after elimination of the etiological source—for example anti-viral therapy in viral hepatitis [3]; however, currently there are no therapies targeting fibrosis directly that treat hepatic fibrosis with undetermined etiologies such as non-alcoholic steatohepatitis (NASH).

Non-alcoholic fatty liver disease (NAFLD) is a chronic liver disease with pathological presence of hepatic steatosis without established cause of hepatic fat accumulation such as alcohol and genetic disorders [4]. NAFLD is a growing health concern in the developed world; prevalence is estimated to be 30% of the population in the United States, with 70–90% of obese and diabetic affected individuals [5–7]. NAFLD also has numerous other co-morbidities such as insulin resistance, type II diabetes, and cardiovascular diseases that increase its clinical significance. NAFLD-related liver cirrhosis is projected to be the leading cause of liver transplantation in the United States by 2020 [6]. NAFLD is often asymptomatic; early detection remains a challenge due to limitation in diagnostic approaches which at present are time consuming and invasive. Therefore diagnosis of NAFLD is often made after significant advancement of diseases such as development of steatohepatitis, fibrosis, and cirrhosis and/or hepatocellular carcinoma. A better understanding of the mechanism behind progression of NAFLD is needed to develop successful therapies for NAFLD.

In development of fibrosis, myofibroblasts are key effector cells that generate the excess extracellular matrix (ECM) in scar tissue. Myofibroblasts are pro-proliferative contractile cells that are characterized by expression of alpha smooth muscle actin (α-SMA) and production of collagen that aids in closing of the wound during the wound healing process. Activated hepatic stellate cells (HSCs) are the primary contributor of the pathogenic ECM production in hepatic fibrosis. Therefore, activation of HSC is a key step in fibrosis. Indeed, depletion of HSCs in murine models has shown remarkable attenuation of both hepatic fibrosis and reduced liver injury and inflammation, suggesting that activated HSCs play an additional role as amplifier of injury in addition to fibrogenesis [8]. Therefore attenuation of HSC activation may be a key pathway in developing novel anti-fibrotic therapeutics.

Thrombospondin I (TSP-1, THBS1) is a large secreted matricellular protein that interacts with a variety of ligands, including receptors, growth factors, proteases, cytokines and structural components of the extracellular matrix [9]. One of its well-known functions is regulating pro-fibrotic cytokine TGF-β1 activity. Specifically, short linear peptide sequence Lys-Arg-Phe-Lys (KRFK) in TSP-1 binds to Leu-Ser-Lys-Leu (LSKL) motif in the latent associated peptide (LAP) of TGF-β1, resulting in disengagement of LAP from TGF-β1 for consequent TGF-β1 activation [10, 11]. TSP-1 null mice display similar but less extreme pheontypes of TGF-β1 deficiency, including multiple organ inflammation and epithelial hyperplasia [12].

In addition to TGF-β1 activation, TSP-1 is also important in numerous cellular functions such as angiogenesis, inflammation, cellular adhesion, migration and growth. TSP-1 interacts with a variety of proteins, including its receptors CD36, CD47 and other proteins of the extracellular matrix—including matrix metalloproteinases and integrins—to fulfill its many functions in the cell in a context-dependent nature. TSP-1 also plays a critical role in ECM homeostasis by directly impacting collagen fibril formation by interacting with collagen, lysyl oxidase precursors and collagen cross linking sites [13].

TSP-1 expression is increased in various human fibrotic diseases. In the liver, the TSP-1 gene THBS1 is a typical signature of cirrhosis resulting from alcoholic fibrosis, non-alcoholic steatohepatitis (NASH) and congenital hepatic fibrosis [14, 15]. Similar gene expression results are also observed in mouse models of liver diseases including diet induced cholestatic hepatic injury via 3, 5-diethoxycarbonyl-1, 4-dihydrocollidine (DDC) administration and hepatotoxic post-necrotic liver fibrosis by carbon tetrachloride (CCl_4_) [14]. A competitive antagonist peptide (LSKL) targets TSP-1’s interaction site with latent TGF-β1 and also attenuates fibrosis in a mouse model of toxin induced liver fibrosis [16] and subarachnoid fibrosis [17]. TSP-1 deficiency in mice has also been observed to be protective against fibrosis in numerous organs such as high fat diet induced muscle [18] and renal fibrosis [19]. LSKL peptide administration immediately after injury also accelerated liver regeneration in mice following partial hepatectomy, suggesting that TSP-1-mediated activation of TGF-β1 also plays a role in not only fibrosis but also liver regeneration [20].

In addition to being highly expressed in fibrotic tissues, TSP-1 is also highly expressed in adipose tissues in rodent models of obesity and in obese human patients [21, 22]. TSP-1 expression in visceral adipose tissue was strongly associated with obesity, insulin resistance and inflammation in human obese patients [22]. In a mouse model of high fat diet induced obesity, TSP-1 also regulated adiposity and metabolic dysfunction by enhancing adipose inflammation and adipocyte proliferation [23]. TSP-1 has been implicated in numerous manifestation of organ dysfunction in diabetes and obesity, such as diabetic cardiomyopathy, diabetic nephropathy and β- cell function in the pancreas [12, 24, 25].

Due to its role in fibrosis and metabolic dysregulation associated with obesity and diabetes, we hypothesized that TSP-1 may be an important modulator in NAFLD/NASH. One of the methods that closely replicate human NASH phenotype is choline deficient L-amino acid defined diet (CDAHFD). The diet is high in fat (60kcal%fat) with limited amount of methionine (0.1%) and lacks choline. Methionine and choline are necessary for hepatic secretion of triglycerides as VLDLs. In addition to a high fat diet, limiting methione or eliminating choline results in impaired lipid export from the liver, which results in progressive development of hepatocellular steatosis, hepatocyte death, inflammation, oxidative stress and fibrosis, similar to typical disease manifestation and progression in human NASH [26].

Here, we report hepato-protective effect of TSP-1 modulation in primary human hepatic stellate cells *in vitro* and in an *in vivo* model of NASH in mice. TSP-1 modulation by knockdown or inhibition attenuated HSC activation measured by α-SMA and Collagen I expression in primary hepatic stellate cells. TSP-1 deficient mice in the choline deficient L-amino acid defined high fat diet (CDAHFD) model of NASH exhibited protection against numerous NASH phenotypic features including decrease in serum lipid levels, inflammation markers and fibrosis compared to WT. Comparison of transcriptomic profiles of TSP-1 null and WT animals suggested that amino acid metabolism, PPARα and fatty acid metabolism pathways are differentially expressed in TSP-1 null mice, suggesting that these pathways may be important in TSP-1 mediated modulation of NAFLD mediated hepatic fibrosis. These findings provide important insight into the role of TSP-1 in context of NAFLD/NASH that may ultimately lead to identification of future anti-fibrotic therapeutics against NAFLD.

## Materials and methods

### Cell culture

Primary human hepatic stellate cells (HSCs) were purchased (Sciencell, Carlsbad, CA) and cultured with Stellate Cell Medium (Sciencell, Carlsbad, CA) in Collagen I coated flasks (Corning, Corning, NY) at 37° C in a humidified atmosphere containing 5% CO_2_. All experiments were performed with cells with passage number less than 8.

### Adenoviral transduction of primary human hepatic stellate cells

HSCs were seeded into collagen coated 96-well plate (Corning, Corning, NY) at density of 10,000 cells/well in stellate cell medium (Sciencell, Carlsbad, CA), incubated for 24 hours then transduced with adenoviral vector containing shRNA against THBS1 (Vector Biolabs, Malvern, PA). Media was aspirated after 24 hours then replaced with fresh media. All experiments were conducted 48 hours after transduction.

### Animals

All procedures involving animals were reviewed and approved by the Pfizer Institutional Animal Care and Use Committee. C57BL/6J and TSP-1 knockout male mice of 5 weeks of age were purchased (The Jackson Laboratory, Bar Harbor, Maine) and were acclimated for 1 week before initiating the diet. Animals were housed and acclimated under 12 hour light-dark cycles with free access to water. At day 0, C57BL/6J mice (The Jackson Laboratory, Bar Harbor, ME) or TSP-1 Knockout mice (The Jackson Laboratory, Bar Harbor, ME, Stock #006141) were provided with either control chow (Research Diets Inc., New Brunswick, NJ, Cat# A08051501) or CDAHFD chow (Choline deficient L-amino acid-defined high fat diet, Research Diets Inc., New Brunswick, NJ, Cat# A06071309) with 10–15 mice were assigned per cohort (Table 1). Bodyweights were measured every 2 weeks. After 12 weeks all groups were sacrificed by CO2 asphyxiation. Serum was collected for liver enzymes and lipid level measurements. Whole livers were weighed and divided into three sections: left lateral lobe (LLL), right lateral lobe (RLL) and right medial lobe (RML). Tissue sections were immersion in 10% neutral buffered formalin fixed for paraffin embedding and slide preparation or snap-frozen in liquid nitrogen for RNA extraction.

**Table 1:** Genes and TaqMan assay IDs.

Gene Symbol	Assay ID
Tgfb	Mm01178820
Tnfa	Mm00443258
Col1a1	Mm00801666
Col3a1	Mm01254476
Gapdh	Mm99999915

### Tissue collection, slide preparation and staining

At necropsy, one section each of LLL, RLL and RML from whole liver was taken and placed into a tissue cassette in 10% neutral buffered formalin for 48 hours and then switched to 70% ethanol until embedded in paraffin. Paraffin-embedded sections were cut and stained with Picrosirius red (PSR) or Hematoxylin and Eosin (H&E) for microscopic assessment by a board-certified veterinary pathologist. PSR stained slides were scanned using a Leica AT2 scanner (Leica Biosystems Inc.). Definiens Tissue Studio image analysis software (Definiens AG, Munich, Germany) was used to measure PSR staining, the areas of PSR staining was extracted from whole slide images of the liver, and the results were exported as the area percentage of PSR staining in whole slide images.

### RNA extraction from liver tissues for RNA-Seq and relative qRT-PCR

RNA extraction from right lateral lobe of the liver was performed with TRIzol (Thermo Fisher Scientific, Waltham, MA) after homogenization with Omni TH tissue homogenizer (Omni International, Kennesaw, GA). Right lateral lobe was chosen because frozen RLL sections were consistently available for RNA extraction across all groups. The quality of the isolated RNA was assessed using Qubit Fluorimeter Qubit 2.0 fluorimeter (Thermo Fisher Scientific, Waltham, MA) and Agilent 2100 Bioanalyzer (Agilent Technologies, Santa Clara, CA), respectively.

### cDNA library construction and sequencing

1 ug of RNA was used for cDNA library construction at Novogene using a NEBNext Ultra Directional RNA library Prep Kit for Illumina (New England Biolab, Ipswich, MA, Cat# E7420S) according to the manufacture’s protocol. Briefly, mRNA was enriched using oligo (dt) beads followed by two rounds of purification and fragmented randomly by adding fragmentation buffer. The first strand cDNA was synthesized using random hexamers primer, after which as custom second-strand synthesis buffer (Illumina), dNTPs, RNase H and DNA polymerase I were added to generate the second-strand (ds cDNA). After a series of terminal repair, poly-adenylation, and sequencing adaptor ligation, the double-stranded cDNA library was completed following size selection and PCR enrichment. The resulting 250–350 bp insert libraries were quantified using a Qubit 2.0 fluorimeter (Thermo Fisher Scientific, Waltham, MA, USA) and quantitative PCR. Size distribution was analyzed using an Agilent 2100 Bioanalyzer (Agilent Technologies, Santa Clara, CA, USA). Qualified libraries were sequenced on an Illumina HiSeq 4000 Platform (Illumina, San Diego, CA, USA) using a paired-end 150 run (2x150 bases). 20M raw reads were generated from each library.

### Pathway enrichment analysis

Illumina HiSeq 4000 sequenced gene profiling were processed and analyzed using QuickRNASeq as described previously [27]. RNA-seq data were deposited in the Gene Expression Omnibus with the accession number GSE120977. The mapped reads were cut off at reads per kilobase per million reads (RPKM) >0.5. LIMMA R package was used in differentially expression analysis [28]. Differentially expressed genes (DEG) were identified with false discovery rate (FDR) <0.05 and log_2_FC >0.58 (1.5 fold change) for enrichment and comparison analysis of biological process ontology, differentially expressed genes were analyzed and visualized in Jomics (http://jomics.org).

### Relative qRT-PCR

10 ng of RNA extracted from liver tissue was used for reverse transcription and hybridization reaction was performed using One step RNA-to-Ct (Thermo Fisher Scientific, Waltham, MA) kit and TaqMan gene expression Assays primers (Thermo Fisher Scientific, Waltham, MA) on ViiA7 Real-Time PCR System (Thermo Fisher Scientific, Waltham, MA) according to manufacturer’s instructions. The list of primers used for the quantitative RT-PCR is listed in Table 1. All values were normalized to GAPDH/Gapdh. All HSC samples were run in three biological replicates with three technical replicates per experiment. All mouse samples were run in 5 biological replicates with two technical replicates per experiment. Fold changes were calculated using 2^-ΔΔCt^ method. The calculated threshold values were determined by maximum curvature and ΔCt was calculated as Ct^control^-Ct^sample^.

### Protein extraction and Western Blot

Cells were washed twice in cold PBS-CMF (Thermo Fisher Scientific, Waltham, MA) then lysed in 1x cell lysis buffer (Cell Signaling Technology, Danvers, MA) supplemented with Complete Protease inhibitor (Roche, Basel, Switzerland). Lysate was agitated for 30 minutes on ice then centrifuged for 15 minutes at 4° C. Lysate supernatant was collected after centrifugation and total protein was measured using BCA Protein Assay Kit (Thermo Fisher Scientific, Waltham, MA). 30 ug of total protein was loaded onto NuPAGE 4–12% Bis-Tris Protein gel (Thermo Fisher Scientific, Waltham, MA) using 1x MES gel running buffer (Thermo Fisher Scientific, Waltham, MA). Post electrophoresis, the gel was blotted onto nitrocellulose membrane using iBlot 2 Gel transfer device (Thermo Fisher Scientific, Waltham, MA) and iBlot2 transfer stacks (Thermo Fisher Scientific, Waltham, MA). The membrane was blocked for an hour in Li-Cor Blocking Buffer–TBS (Li-Cor Biosciences, Lincoln, Nebraska) and incubated overnight at 4 C in primary antibody diluted 1:1000 in blocking buffer (anti-human THBS1 Cat# 37879, GAPDH Cat#5174 were both from Cell Signaling Technology, Danvers, MA). After overnight incubation, the membrane was washed 3 times with 5 minutes of agitation using TBS supplemented with 0.1% Tween20 (Sigma Aldrich, Saint Louis, MO). The washed membranes were incubated at room temperature for an hour with secondary antibody (Li-COR IRDye 800 CW goat anti-rabbit IgG, Licor Biosciences, Lincoln, Nebraska, Cat# 925–32211) at 1:15,000 dilutions with blocking buffer. After incubation, the membranes were washed 3 times using TBS supplemented with 0.1% Tween20 (Sigma Aldrich, Saint Louis, MO) and final wash was with PBS-CMF (Thermo Fisher Scientific). Stained and washed membrane was read on Li-Cor Odyssey CLx imaging system (Li-Cor Biosciences, Lincoln, Nebraska). Densitometries of the blots were quantitated using Li-Cor software.

### Immunofluorescence

HSCs were seeded overnight in Viewplate Collagen I coated 96 well plates (Perkin Elmer, Waltham, MA) with Stellate Cell Media (Sciencell, Carlsbad, CA). Following day, HSCs were serum starved in stellate cell media supplemented with 0.3% FBS (Sciencell, Carlsbad, CA) overnight followed by TGF-β1 (R&D Systems, Minneapolis, MN) treatment for 48 hours. For peptide inhibition experiments, HSCs were treated with peptides 1.5 hours prior then treated with TGF-β1. After 48 hours, HSCs were fixed using ice-cold solution made of 50% acetone (Sigma- Aldrich, Saint Louis, MO) and 50% methanol (Sigma- Aldrich, Saint Louis, MO). The cells were blocked with PBS-CMF supplemented with 5% goat serum (Thermo Fisher Scientific, Waltham, MA) and 0.5% Triton-X (Sigma- Aldrich, Saint Louis, MO) for 30 minutes. Following blocking the cells were stained with primary (α-SMA: Sigma Aldrich, Saint Louis, MO, Cat# A5228; Collagen I: Abcam, Cambridge, MA, Cat #ab6308) antibodies and secondary antibodies (goat anti-mouse IgG Alexa 568, Thermo Fisher Scientific, Waltham, MA, Cat# A11-004) diluted 1:1000 in PBS-CMF supplemented with 2.5% goat serum (Thermo Fisher Scientific, Waltham, MA) and 0.5% Triton-X (Sigma- Aldrich, Saint Louis, MO) for 1 hour each. Washes were performed 3 times between each step using PBS-CMF (Thermo Fisher Scientific, Waltham, MA). After staining, the cells were scanned on Operetta High Content Imaging System (Perkin Elmer, Waltham, MA). Mean fluorescence intensity per well was quantitated using Harmony Phenologic (Perkin Elmer, Waltham, MA) software and images were exported from Volocity (Perkin Elmer, Waltham, MA) software. All measurements were done in triplicates with 3 independent experiments.

### Statistical analysis

Data are presented as mean +/- standard error of the mean (SEM) with analysis performed using student’s t test or analysis of variance (ANOVA) using GraphPad Prism version 7.04. Statistical analysis was performed using student’s t test or ordinary one-way ANOVA using multiple comparison (Sidak’s or Tukey’s multiple comparison). Significance was set at p<0.05.

## Results and discussion

**Fig 1:**
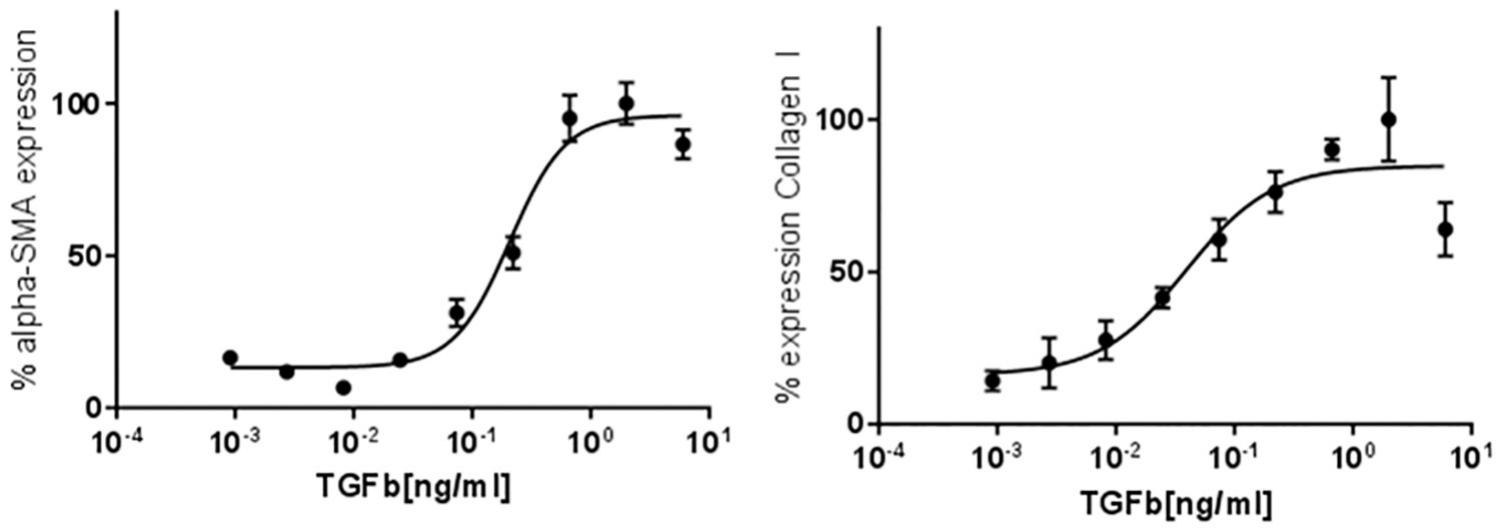
TGF-β1 dose response with α-SMA and Collagen I expression in primary human HSCs. HSCs were treated for 48 hours with 9–0.001 ng/ml change to lowest to highest TGF-β1 and then fixed and stained for α-SMA and Collagen I. Percent value is based on maximal response from addition of TGF-β1. EC_50_ α-SMA expression: 200 pg/ml TGF-β1; EC_50_ Collagen I expression: 50 pg/ml TGF-β1. % expression values were calculated by defining zero as the smallest value in each data set and one hundred as the largest value in each data set.

**Fig 2:**
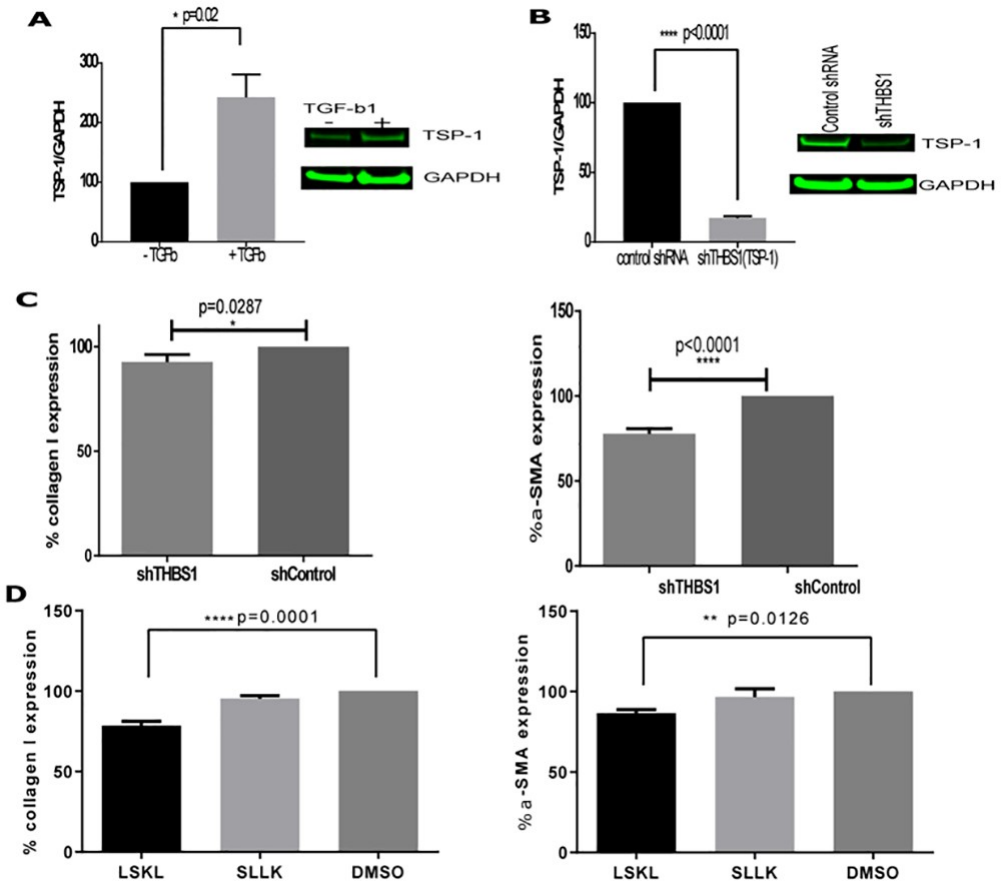
Peptide-mediated inhibition or knockdown of TSP-1, attenuates markers of primary human HSC activation into myofibroblasts. A. Primary human HSCs activated with 1 μg/ml TGF-β1 for 48 hours show increased expression of TSP-1 by Western Blot analysis. Bar graphs represent densitometry measurements, error bars show standard error of the mean (SEM). TSP-1/GAPDH expression was normalized to values from cells treated with vehicle (0.01% DMSO). B. Expression of TSP-1 is significantly reduced in TGF-β1 activated primary human HSCs transduced with adenoviral particles carrying shRNA against transcribed gene for TSP-1 (shTHBS1) compared to HSCs transduced with control shRNA adenovirus. Cells were transduced at multiplicity of infection (MOI) of 300 for 48 hours prior to treatment with TGFβ then harvested after 48 hours. Bar graph represent densitometry measurements, error bars show SEM. TSP-1/GAPDH was normalized to cells transduced with adenovirus carrying control shRNA. C. TSP-1 knockdown by shRNA transduction in primary human HSCs attenuate TGF-β1 mediated expression of markers of fibrosis, alpha smooth muscle actin (α-SMA) and Collagen I measured by immunofluorescence. HSCs were transduced with adenoviral shTHBS1 (gene for TSP-1) for 48 hours then treated with TGF-β1 for 48 hours prior to fixation for immunofluorescence, n = 3 per treatment, per experiment. Error bars represent SEM; % expression is normalized to cells transduced with control shRNA. D. Treatment with inhibitory peptide against TGF-β1 activating motif (LSKL) in TSP-1 results in attenuation of TGF-β1 mediated expression of markers of fibrosis, α-SMA and Collagen I. n = 3 per treatment, per experiment. Peptide with scrambled sequence (SLLK) does not significantly (n.s) impact expression of TGF-β1-induced α-SMA or Collagen I. Error bars represent SEM; % expression is normalized to cells treated with vehicle (0.1% DMSO). All presented data are representative of at least 3 separate experiments. Statistical analysis was performed using GraphPad Prism v 7.04 with either student’s t test or oneway ANOVA Tukey’s multiple comparison tests. P value was considered significant at <0.05.

The activation of hepatic stellate cells (HSCs) to myofibroblasts is central to development of pathogenic fibrosis. To understand the role of TSP-1 in HSC activation, we utilized primary human hepatic stellate cells as our *in vitro* model system. HSC activation and differentiation into myofibroblasts can be measured by TGF-β1 dose dependent increase in α-SMA and Collagen I expression (Fig 1). We first confirmed that TSP-1 is induced by TGF-β1 treatment in our model system (Fig 2A) by Western Blot analysis. Quantification of densitometry determined that TSP-1 expression was significantly increased (242.6%, p = 0.02) after primary human HSCs were treated for 48 hours with 1 μg/ml TGF-β1 (at a concentration >EC_100_ for α-SMA and Collagen I expression) compared to vehicle treated HSCs. To understand the impact of TSP-1 in HSC activation, we performed a knockdown experiment with *THBS1* (gene for TSP-1) shRNA and measured markers of myofibroblast activation, α-SMA and Collagen I. Primary HSCs were transduced with *THBS1* shRNA carrying adenoviral particles for 48 hours followed by 1 μg/ml TGF-β1 treatment for another 48 hours. A control set of HSCs were transduced with scramble shRNA adenoviral particles. Following the transduction and TGF-β1 induced activation, Western Blot analysis confirmed successful knockdown (83%, p<0.0001) of TSP-1 protein expression (Fig 2B) compared to the control cells transduced with scramble shRNA. We then measured the impact of TSP-1 knockdown on activation of primary human HSCs by measuring Collagen I and α-SMA expression by immunofluorescence at EC_50_ values of TGF-β1 mediated increase in Collagen I or α-SMA by immunofluorescence (Fig 1). Next, HSCs were first transduced for 48 hours with either sh*THBS1* or scramble shRNA carrying adenovirus then treated with 50 pg/ml (for Collagen I measurement) or 200 pg/ml (for α-SMA measurement) of TGF-β1, EC_50_ concentrations of TGF-β1 for inducing expression of Collagen I and α-SMA. Cells transduced with sh*THBS1* adenovirus had attenuated α-SMA (75.5%, p<0.0001) and to a less extent Collagen I (92.6%, p = 0.0287) expression in TGF-β1 activated primary human HSCs (Fig 2C) than cells transduced with scramble shRNA. These results from TSP-1 knockdown experiment suggest that TSP-1 is important in TGF-β1-mediated primary human HSC activation.

TSP-1 is an important regulator of TGF-β1 activation in fibrosis. We examined whether the attenuation observed in the knockdown experiment may be attributed to TSP-1 mediated TGF-β1 activation. TGF-β1 is secreted as a latent complex that requires activation in order to bind to its receptor, which triggers production of more TGF-β1, creating a positive feedback loop. To examine whether the inhibition of TSP-1 mediated TGF-β1 activation impacts HSC activation, HSCs were treated with 3μM of inhibitor peptide (LSKL) that prevents the activation of secreted TGF-β1 followed by recombinant TGF-β1 (200 pg/ml for α-SMA and 50 pg/ml for Collagen I measurement). Scrambled peptide (SLLK) or vehicle (0.1% DMSO) treatments were also tested as controls. HSCs were fixed and stained for α-SMA and Collagen I after 48 hours and expression was quantified by immunofluorescence. Similar to our observation with TSP-1 knockdown in primary HSCs, there was a significant decrease in both α-SMA (86.7%, p = 0.0126) and Collagen I (78.6%, p<0.0001) expression in LSKL treated cells compared to cells that were treated with vehicle 0.1% DMSO (Fig 2D). Treatment with scrambled peptide did not result in significant decrease in either marker of fibrosis. Therefore, attenuation of α-SMA and Collagen I observed in TSP-1 knockdown may be due to TSP-1’s role in activation of latent TGF-β1 as demonstrated by peptide mediated inhibition of TSP-1 mediated latent TGF-β1 activation.

Next, we explored whether modulation of TSP-1 impacts liver fibrosis in an *in vivo* model of a progressive fibrotic disease of the liver, non-alcoholic steatohepatitis (NASH). Briefly, 10–15 male C57/BL6J (wild type, WT) or TSP-1 knockout (TSP1 KO) mice were obtained and housed under 12h light-dark cycles with free access to water and control or CDAHFD chow (Table 2). Body weight was measured every 2 weeks. After 12 weeks, animals were euthanized for analysis. Serum samples were collected, and livers were harvested, weighed, and divided into three different sections (right medial lobe, left lateral lobe, and right lateral lobe) that were immersion-fixed in neutral buffered formalin for slide preparation or snap frozen in liquid nitrogen.

**Table 2:** Four groups of mice in the study.

Group ID	N (# of mice)	Diet	Genotype	Study duration
NDWT	10	Control	WT	Weeks 1–12
CDAHFDWT	15	CDAHFD	WT	Weeks 1–12
NDKO	10	Control	TSP-1 KO	Weeks 1–12
CDAHFDKO	15	CDAHFD	TSP-1 KO	Weeks 1–12

NDWT: Control diet fed wild type mice. CDAHFDWT: CDAHFD fed wild type mice. NDKO: Control diet fed TSP-1 KO mice. CDAHFDKO: CDAHFD fed TSP-1 KO mice.

**Fig 3:**
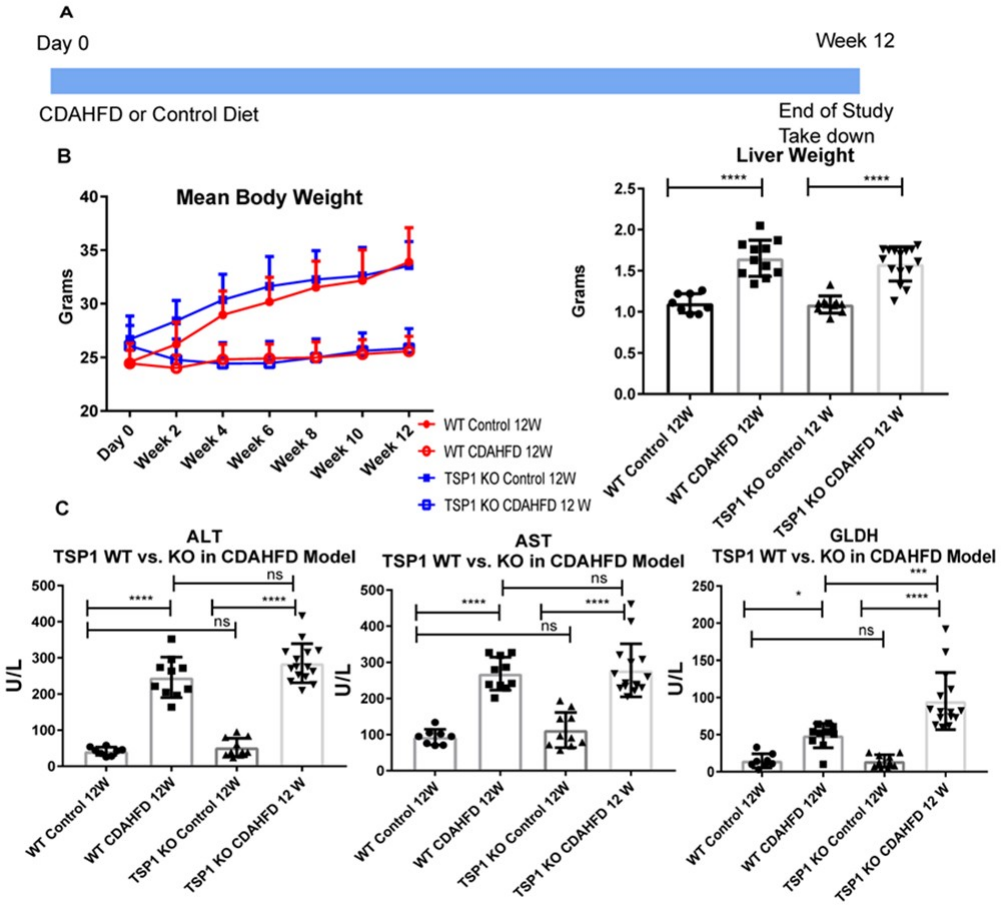
Study design and body weight, liver weight and liver function comparison of CDAHFD vs normal diet fed TSP-1 KO and Wild type mice. A. Study design outline. B. Both wild type (WT) and TSP-1 null mice (TSP-1 KO) mice fed choline deficient l-amino acid defined diet (CDAHFD) did not show increase in body weight with increase in time. Both WT and TSP-1 null mice fed control diet increased similarly in body weight with increase in time with heaviest weight at week 12. Both TSP-1 null and WT mice fed CDAHFD had significantly increased liver:body weight ratio and liver weight compared to mice fed control diet. C. Alanine aminotransferase (ALT) and Aspartate aminotransferase (AST) and glutamate dehydrogenase (GLDH) of CDAHFD vs. control diet fed mice were significantly increased in both WT and TSP-1 null groups. No significant difference was observed between TSP-1 null and WT groups fed control diet for ALT, AST and GLDH. In CDAHFD fed mice, TSP-1 null mice had significantly higher GLDH than WT. Each point represents a single animal. Statistical analysis are for each genotype group (TSP-1 KO or WT) or for each diet group (CDAHFD or control), using one way ANOVA Tukey’s multiple comparison tests. Error bars represent standard error of the mean (SEM). P value was considered significant at <0.05. **** p<0.0001, ***p = 0.0003, *p = 0.0325.

After 12 weeks on normal diet, TSP-1 null cohort compared to WT cohort did not differ in either mean total body or liver weight (Fig 3B). Similarly, comparison of TSP-1 null and WT mice fed CDAHFD did not differ significantly in total body and liver weight. However, there was a significant difference between cohorts fed CDAHFD vs. control diet in both genotypes. CDAHFD fed mice maintained mean body weight throughout the 12 weeks of study whereas the mean body weight of control diet fed mice increased. In contrast, the liver weight of CDAHFD mice were significantly increased compared to the control diet fed mice. These observations are consistent with a previous report [29]. We also observed that levels of ALT, AST and GLDH were significantly increased (****p<0.0001) in both CDAHFD fed WT and TSP-1 null groups (Fig 3C). However, serum aminotransferase levels are not significantly different between TSP-1 null and WT in both diet cohorts, except GLDH levels were elevated (p = 0.0003) in TSP-1 null mice fed CDAHFD compared to WT mice fed CDAHFD.

**Fig 4:**
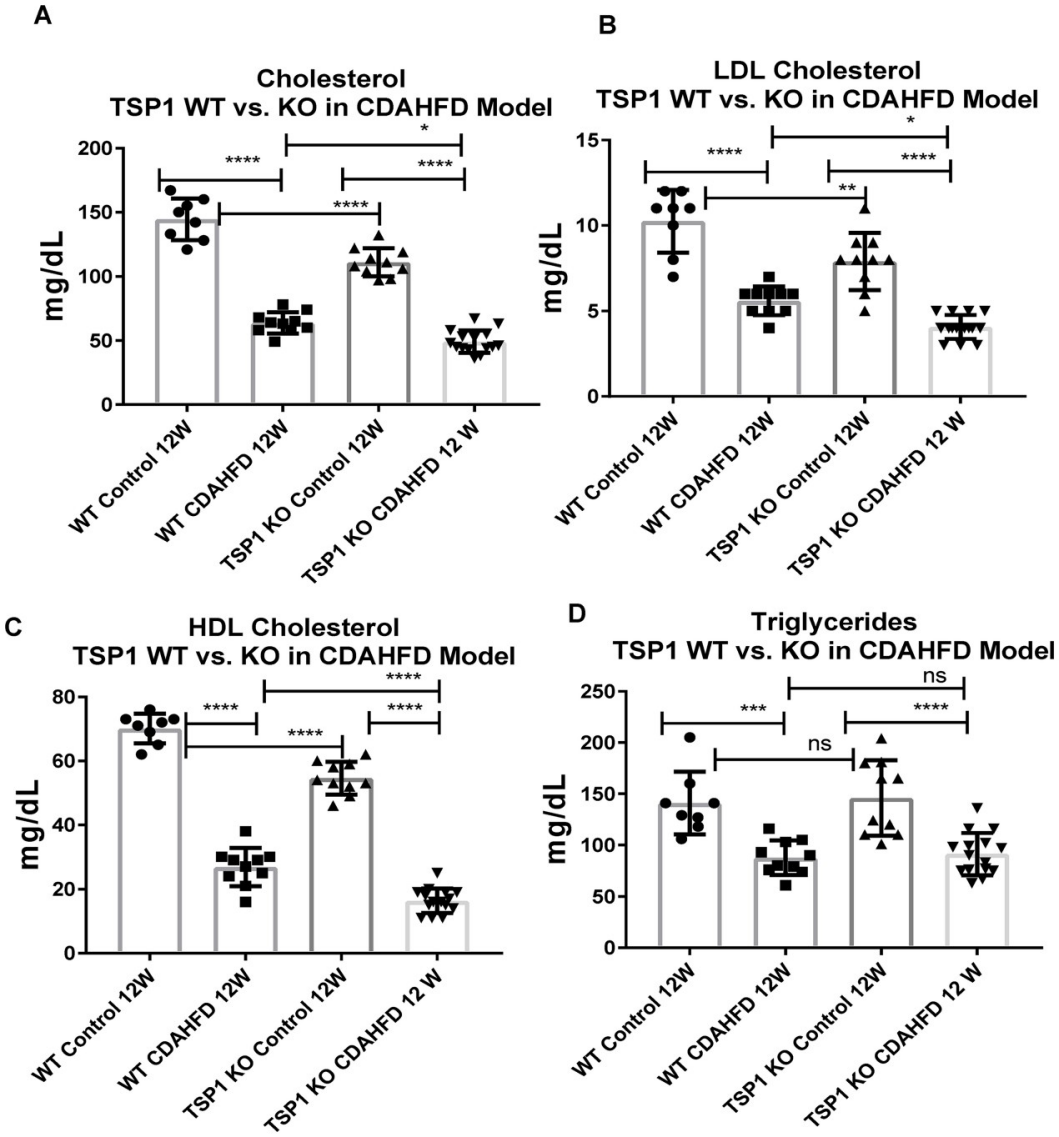
Lipid profile comparison of CDAHFD vs Normal diet fed TSP-1 null (TSP-1 KO) and Wild type mice at 12 weeks. Comparison of serum levels of A. total cholesterol, B. Low density lipoprotein (LDL) cholesterol C. High density lipoprotein (HDL) cholesterol and D. Triglycerides measurements at week 12 of CDAHFD or control diet fed mice in both TSP-1 null and WT mice. Comparison between WT vs TSP-1 null animals demonstrate significantly reduced serum cholesterol (total, LDL and HDL) levels in TSP-1 null vs WT in both control diet and CDAHFD fed cohorts. Each point represents a single animal. Error bars represent standard error of mean (SEM). Statistical analysis are for each genotype group (TSP-1 KO or WT) or for each diet group (CDAHFD or control), using one way ANOVA Tukey’s multiple comparison tests ****p <0.0001 for all graphs, *p = 0.0115 for cholesterol, ** p = 0.0018, p = 0.0241 for LDL cholesterol, *** p = 0.0007 for triglycerides, ns = no significance between compared groups.

We then examined measurements of various lipid levels in the serum. Similar to serum liver enzyme levels, there were significant differences between CDAHFD vs control diets (p<0.0001) in serum total cholesterol, low density lipoprotein (LDL) cholesterol, high density lipoprotein (HDL) cholesterol and triglyceride levels in both WT and TSP-1 null genotypes. However, unlike total body weight, liver weights and serum liver enzyme level comparisons, TSP-1 null compared to WT had significantly reduced total, LDL and HDL cholesterol in both control or CDAHFD diets (Fig 4A and 4B). No significant difference was observed between TSP-1 null mice and wild type mice in both diet groups in triglyceride levels.

**Fig 5:**
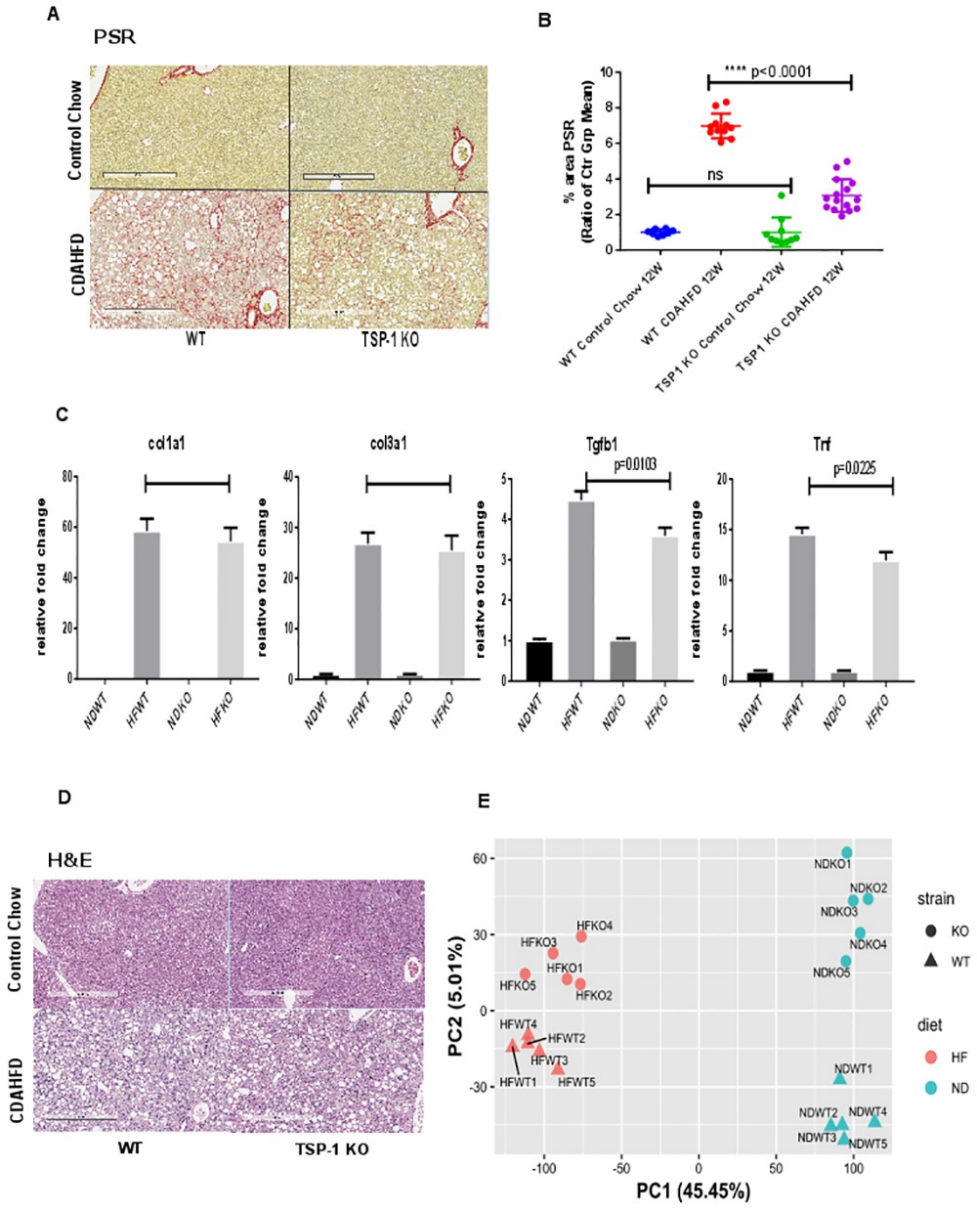
TSP-1 null mice are protected against CDAHFD induced liver fibrosis. A. Representative images of mouse liver sections stained with Picrosirius red (PSR). B. TSP-1 KO mice fed CDAHFD had significantly lower PSR+ area compared to WT mice fed CDAHFD (**** p<0.0001). The graphs were plotted as ratio of control group mean (using group mean of concurrent age matched control chow values for PSR. Statistical analysis was done using ordinary one-way ANOVA using multiple comparison (Sidak’s multiple comparison test) of selected pairs to compare control diet and CDAHFD groups from WT and TSP-1 null genotypes (total 2 selected pairs). C. Relative qRT-PCR analysis of collagen and inflammation markers from liver mRNA. Error bars represent SEM. Significance at p<0.05 NDWT: Control diet fed wild type mice. HFWT: CDAHFD fed wild type mice. NDKO: control diet fed TSP-1 null mice. HFKO: CDAHFD fed TSP-1 null mice. Relative fold change normalized to equivalent genotype fed control diet. Statistical analysis was done using ordinary one-way ANOVA using Tukey’s multiple comparison test with GraphPad Prism v. 7.04. D. Representative H&E stains of mouse liver sections. Bar represents 500 μm. E. Principal Component Analysis (PCA) of liver RNA-seq gene expression data. Each data point represents individual mice. Difference in genotype is represented by shape (circle: TSP-1 null or KO, triangle: wild type or WT). Difference in diet is represented by color (pink: CDAHFD fed or HF, blue: control diet fed, or ND). PCA analysis was performed by build-in R function prcomp() on all genes and the plot was generated by R packages ggplot2 and ggrepel.

We then performed histologic analysis for hepatic steatosis and fibrosis by Picrosirius red (PSR) and hematoxylin and eosin (H&E) (Fig 5A and 4D). Importantly, quantification of percent PSR positive area revealed that TSP-1 null cohort had significantly less collagen and therefore reduced liver fibrosis (Fig 5B) than wild type. To determine whether the decreased fibrosis in TSP-1 null CDAHFD fed mice was due to decreased collagen production, we performed relative qRT-PCR to measure relative mRNA expression of Col1a1 and Col3a1. In addition, we also measured relative mRNA levels of pro-inflammatory markers tumor necrosis factor (Tnf) and transforming growth factor beta 1 (Tgfb1) to determine the effect of TSP-1 deficiency on liver inflammation. Gapdh, with constant Ct values across the samples, was used as reference gene to quantitate relative expression. We observed relative expression of Tnf and Tgfb1 were subtly but significantly decreased in CDAHFD fed TSP-1 null mice (Fig 5C, Table 1). There was no significant difference in the expression of col1a1, col3a1, Tnf and Tgfb1 between TSP-1 null and WT mice fed control diet. (Fig 5C, Table 1).

To better understand the gene expression profiles in the various cohorts, we performed an RNA-seq based transcriptomic analysis of poly (A) + mRNA extracted from right lateral lobe (RLL) of liver from 5 mice randomly selected from each group. Right lateral lobe was chosen because frozen RLL sections were consistently available for RNA extraction across all groups. On average, 86% of raw sequenced reads were uniquely mapped to the mouse reference genome and 88% of the uniquely mapped reads were in genomic exon regions. The mapped reads were cut off at reads per kilobase per million reads (RPKM) >0.5. LIMMA R package was used in differentially expression analysis. Differentially expressed genes (DEGs) were determined by false discovery rate threshold (FDR) <0.05 and genes with fold change greater than 1.5 (log_2_FC>0.58). Principal component analysis (Fig 5E) identified the expression pattern of samples into four groups: NDWT, NDKO, HFWT, and HFKO (NDWT: Control diet fed wild type mice. HFWT: CDAHFD fed wild type mice. NDKO: control diet fed TSP-1 null mice. HFKO: CDAHFD fed TSP-1 null mice). We observed that between the groups that were fed control diet, TSP-1 null mice have distinct expression pattern compared to the wild type (WT) mice. However, this difference between genotypes is reduced in comparison of WT mice and TSP-1 null mice fed CDAHFD. Finally, we also observed greater numbers of DEGs in diet comparison than genotype comparisons (Table 3).

**Table 3:** Summary of DEG analysis based on diet or genotype.

Comparison	Cohort	DEG	Upregulated	Downregulated	Total
Diet	TSP-1 null	CDAHFD/control	2894	1631	4525
Diet	WT	CDAHFD/control	2894	1960	4854
Genotype	control diet	TSP-1 null/WT	288	251	539
Genotype	CDAHFD	TSP-1 null/WT	43	27	70

**Fig 6:**
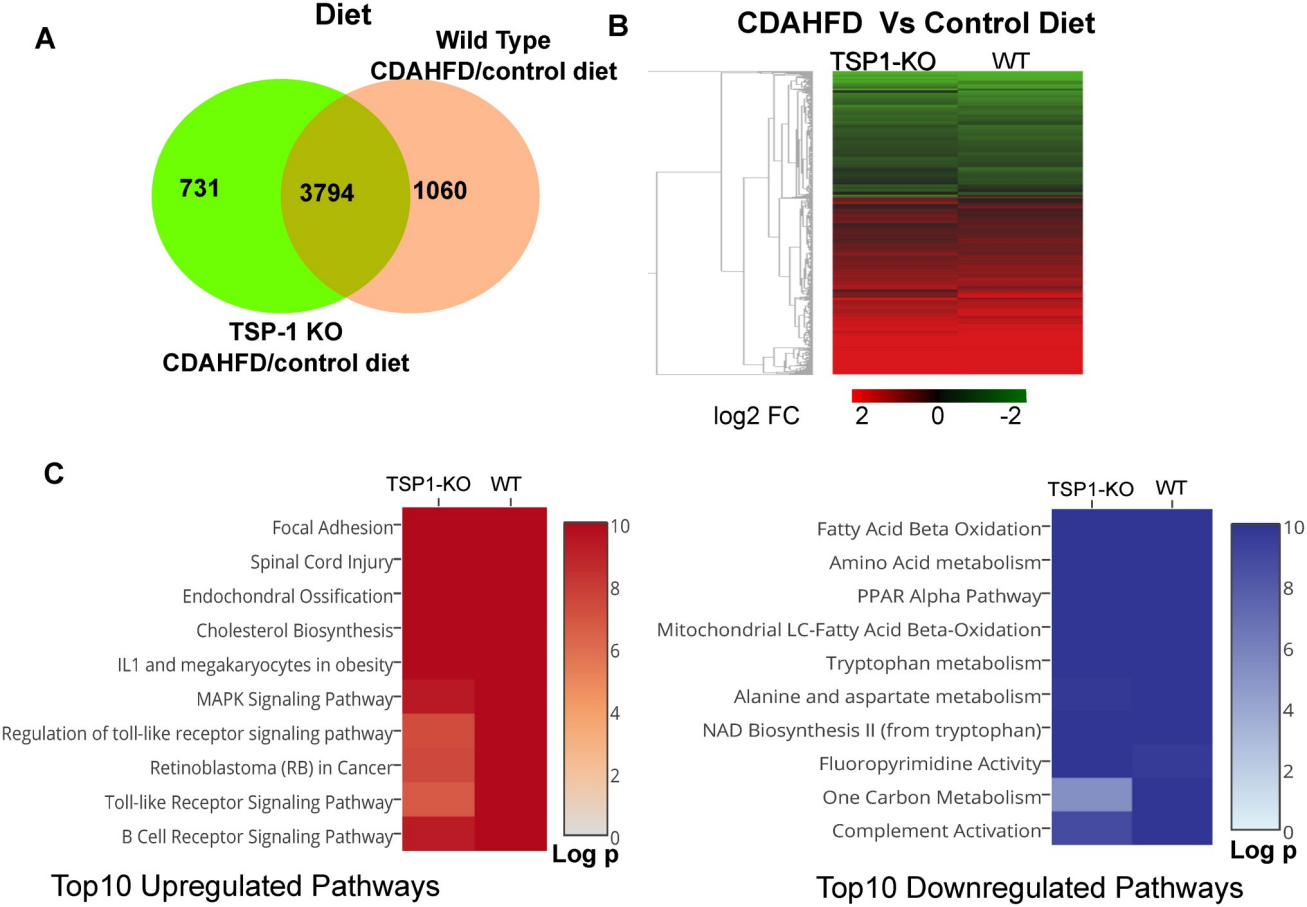
Transcriptomic analysis to compare the impact of CDAHFD vs. control diet in gene expression profiles of TSP-1 KO and WT mice. A. Venn diagram of differentially expressed genes (DEGs) from CDAHFD fed vs control diet fed TSP-1 null mice and WT mice. B. Heat map of fold change (log_2_FC) in DEGs (red: upregulated, green: downregulated) from genes in Fig 5A. C. Heat map with top 10 upregulated (in red) and downregulated (in blue) pathways by WikiPathways. DEGs were defined by FDR>0.05 and >1.5 fold change comparing CDAHFD vs. control diet.

To understand the impact of CDAHFD diet, we analyzed the gene expression changes in TSP-1 null and WT mice fed CDAHFD vs. control diet. Venn analysis of the DEGs from TSP-1 null and WT cohorts revealed that the transcriptomic profiles of both comparisons were very similar, with 3794 DEGs overlapping between the comparisons with 731 DEGs unique to TSP-1 null cohorts and 1060 unique WT cohorts (Fig 6A). Hierarchical clustering and heat map visualization of all DEGs based on fold change demonstrate that the directionality of gene expression changes in TSP-1 null and WT animals are very similar (Fig 6B). WikiPathways Analysis [30, 31] of top ten up- and down-regulated pathways further confirmed that CDAHFD fed TSP-1 null and WT mice have many coinciding pathways with similar low adjusted p-values (Fig 6C). Some of these pathways that were upregulated include: extracellular matrix reorganization, metabolic dysregulation, injury/inflammation and proliferation such as focal adhesion, spinal cord injury, endochondral ossification, cholesterol biosynthesis, IL-1 and megakaryocytes in obesity, MAPK signaling pathway and B cell receptor signaling pathways (Fig 6C). A subtle difference was noted between TSP-1 null and WT mice for pathways relevant to regulation of toll like receptor signaling pathway and retinoblastoma (RB) in cancer. A similar pattern was observed in comparison of downregulated pathways with similar adjusted p values in fatty acid beta oxidation, amino acid metabolism, PPARα pathway, one carbon metabolism and complement activation pathways. Overall the transcriptomic profiles of CDAHFD fed TSP-1 and WT mice were similar with overlapping profiles indicative of pathological disruption, following the trend outlined earlier in total liver:body weight, liver weight, liver enzymes and histopathological analysis comparison.

**Fig 7:**
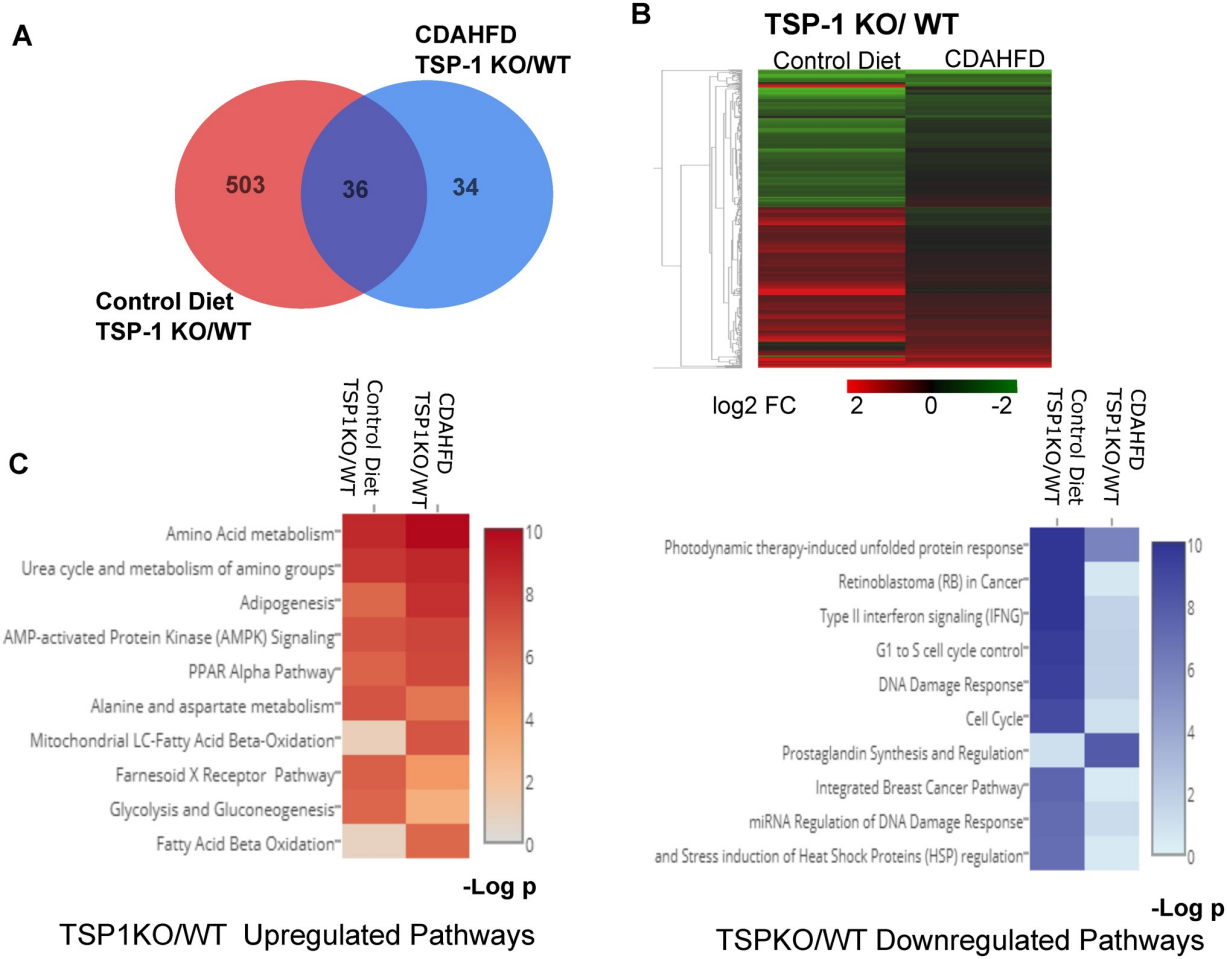
Transcriptomic analysis to compare the impact of TSP-1 KO vs. wild type genotype in CDAHFD vs. control diet fed mice. A. Venn diagram of differentially expressed genes (DEGs) from TSP-1 KO vs WT mice fed CDAHFD or control diet. B. Hierarchical clustering and heat map of DEGs based on log_2_FC values C. Heat map with top 10 upregulated (in red) and downregulated (in blue) pathways by WikiPathways. DEGs were defined by FDR<0.05 and fold change >1.5 (log_2_FC>0.58) comparing TSP-1 null vs. WT mice.

We then analyzed the impact of different genotypes on liver transcriptomic profiles in both control diet and CDAHFD groups. In these comparisons, gene expression from WT mice were used to define differentially expressed genes in TSP-1 null mice. Venn analysis of DEGs from both control diet and CDAHFD group revealed that there are only 36 DEGs that overlap in normal diet and CDAHFD (Fig 7A) in TSP-1 null mice. There are 34 unique DEGs in CDAHFD whereas 503 unique DEGs were identified in control diet (Fig 7A). Small number of DEGs (Table 3, 70 total DEGs) with CDAHFD suggest that the liver transcriptomic profiles of both TSP-1 null and WT animals are heavily driven by CDAHFD and consequent pathogenesis, rather than by genotype. However, greater number of DEGs defined (Table 3, 539 total DEG) from control diet fed TSP-1 null mice demonstrates that under normal conditions, there are notable differences between transcriptomic profiles of TSP-1 null mice and WT mice. Furthermore, similarities in the top ten up- and down-regulated pathways identified by Wiki Pathway Analysis between the two comparisons also confirm that the transcriptomic gene signatures driven by the TSP-1 null genotype is maintained in both control and CDAHFD fed animals (Fig 7C). The top ten upregulated pathways identified in TSP-1 null mice were pathways relevant to amino acid metabolism, PPARα pathway and fatty acid metabolism. The top ten downregulated pathways identified in TSP-1 null mice were pathways relevant to retinoblastoma (RB) in cancer, cell cycle pathways and stress and injury mediated pathways.

**Fig 8:**
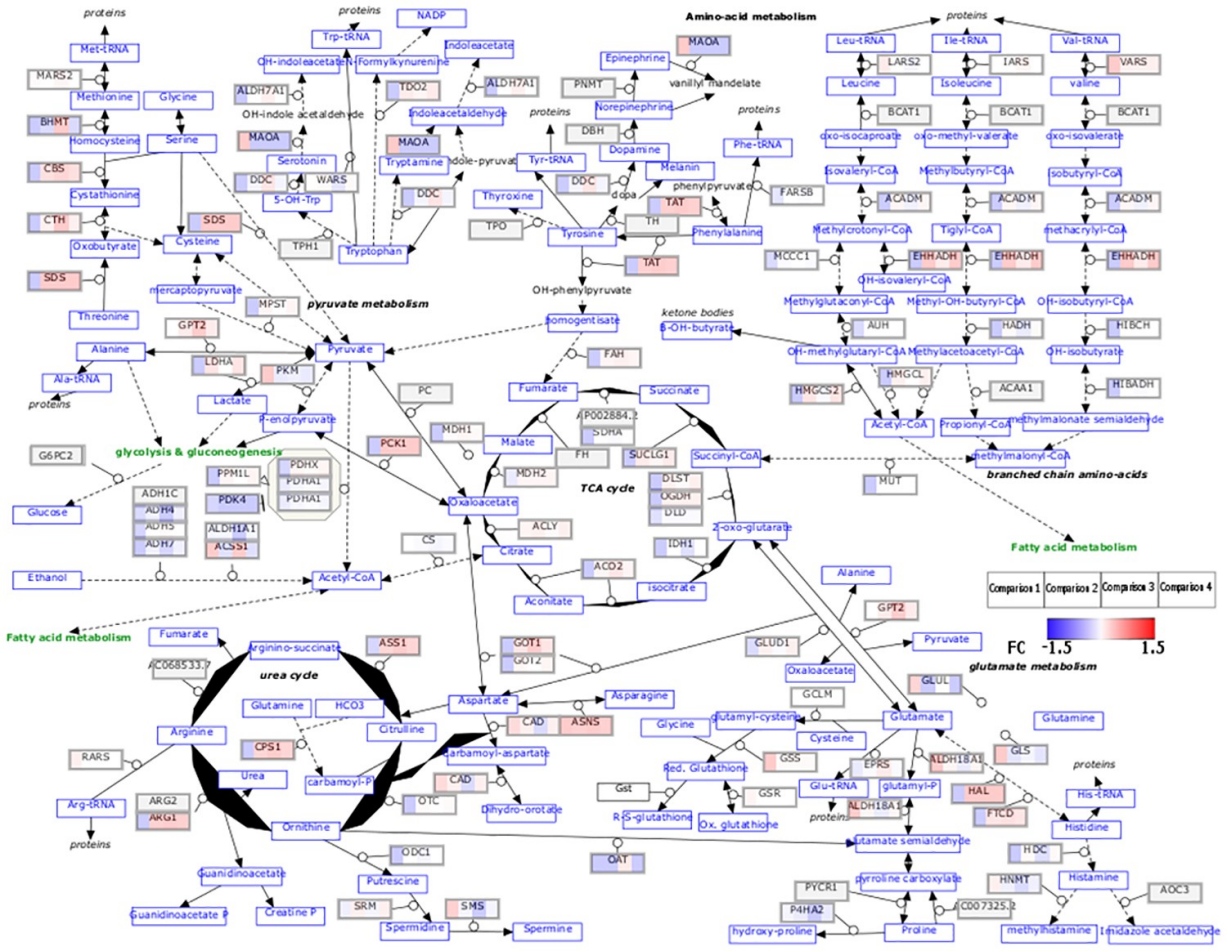
Visualization of log_2_FC values of genes identified in the four comparisons in amino acid metabolism pathway by WikiPathways. Comparison 1 is WT: CDAHFD/control. Comparison 2 is TSP-1 null: CDAHFD/control. Comparison 3 is control diet: TSP-1 null/WT. Comparison 4 is CDAHFD: TSP-1 null/WT.

**Fig 9:**
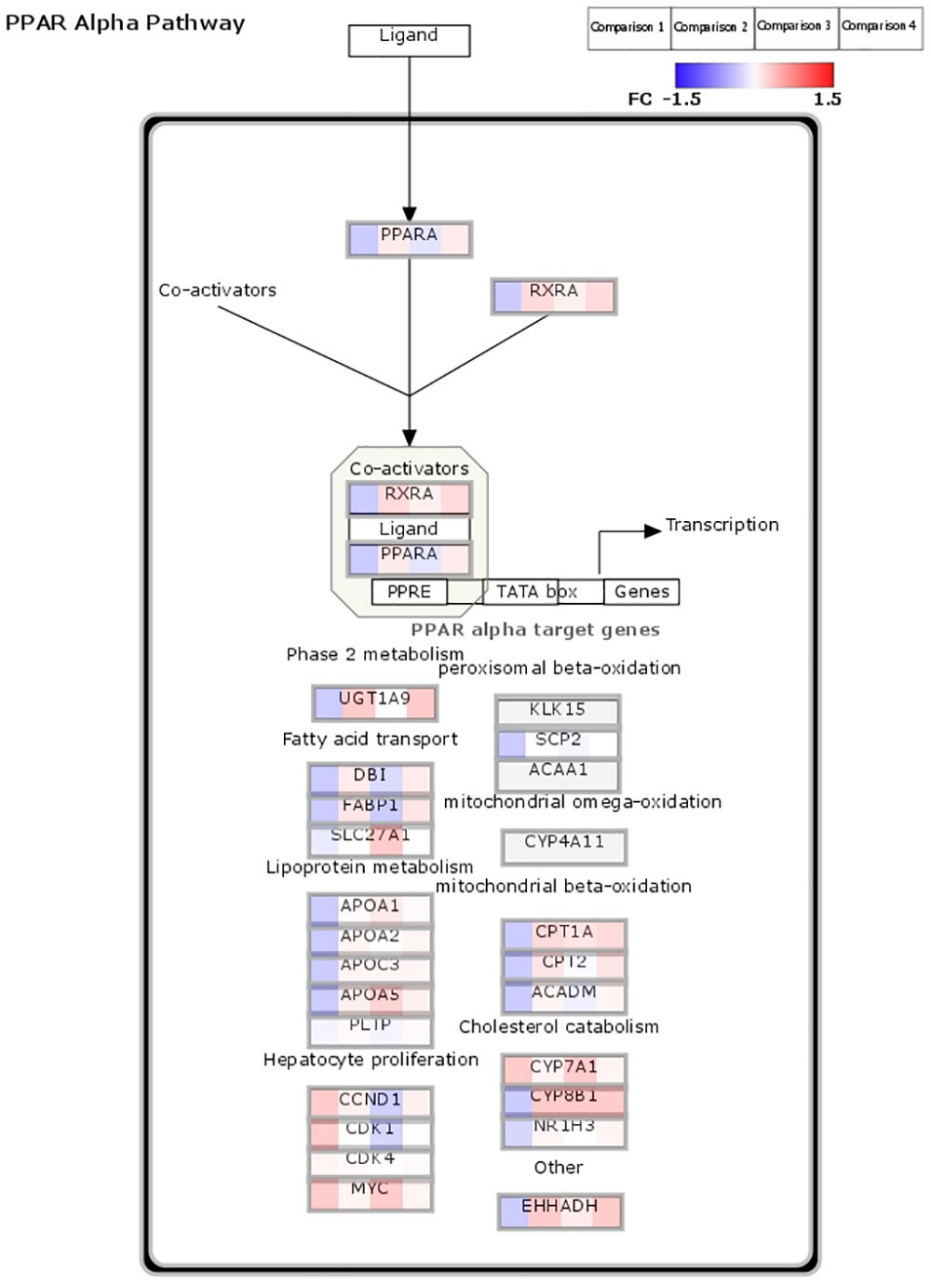
Visualization of log_2_FC values of genes identified in the four comparisons in PPARα pathway by WikiPathways. Comparison 1 is WT: CDAHFD/control. Comparison 2 is TSP-1 null: CDAHFD/control. Comparison 3 is control diet: TSP-1 null/WT. Comparison 4 is CDAHFD: TSP-1 null/WT. FC: fold change.

To identify the pathways that were modulated by TSP-1 that may have contributed to observed decreased hepatic fibrosis in CDAHFD fed TSP-1 null mice, we then identified pathways that were up- or down-regulated by CDAHFD that were reversed in the genotype comparison (Tables 3 and 4). We identified PPARα pathway and amino acid metabolism pathways as pathways that were downregulated by CDAHFD in both genotype groups yet upregulated in TSP-1 KO mice compared to WT mice in both diet conditions. We also mapped the fold change values of specific genes in the amino acid metabolism pathway (Fig 8) and PPARα pathway (Fig 9) as defined by WikiPathways to visualize the specific genes and their place in the pathway. In addition, we also identified fatty acid beta oxidation as a pathway similarly downregulated by CDAHFD in both genotype groups but the pathway was upregulated only in CDAHFD TSP-1 KO mice, suggesting that it is a pathway that is uniquely diet-induced in TSP-1 null mice. In reverse, the Retinoblastoma (RB) cancer pathway was upregulated by CDAHFD but was repressed in wild type but not TSP-1 null mice suggesting that it was likely not a pathway that contributed to the anti-fibrotic effect.

**Table 4:** Summary of pathways of interest identified by WikiPathways.

**Comparison**	**Pathway**	**Direction**	**# of genes**	**-log P**
WT: CDAHFD/control	PPARα Pathway	Down	12	23.7
TSP-1 null: CDAHFD/control	PPARα Pathway	Down	8	14.3
Control diet: TSP-1 null/WT	PPARα Pathway	Up	7	6.4
CDAHFD: TSP-1 null/WT	PPARα Pathway	Up	5	7.5
**Comparison**	**Pathway**	**Direction**	**# of genes**	**-log P**
WT: CDAHFD/control	Amino acid metabolism	Down	17	16.7
TSP-1 null: CDAHFD/control	Amino acid metabolism	Down	19	24.8
Control diet: TSP-1 null/WT	Amino acid metabolism	Up	17	8.8
CDAHFD: TSP-1 null/WT	Amino acid metabolism	Up	12	12.2
**Comparison**	**Pathway**	**Direction**	**# of genes**	**-log P**
WT: CDAHFD/control	Fatty acid Beta Oxidation	Down	14	25.6
TSP-1 null: CDAHFD/control	Fatty acid Beta Oxidation	Down	8	12
Control diet: TSP-1 null/WT	Fatty acid Beta Oxidation	n/a	3	0.8
CDAHFD: TSP-1 null/WT	Fatty acid Beta Oxidation	Up	5	6.2
**Comparison**	**Pathway**	**Direction**	**# of genes**	**-log P**
WT: CDAHFD/control	Retinoblastoma (RB) in Cancer	Up	23	7.5
TSP-1 null: CDAHFD/control	Retinoblastoma (RB) in Cancer	Up	27	11.4
Control diet: TSP-1 null/WT	Retinoblastoma (RB) in Cancer	Down	19	11.5
CDAHFD: TSP-1 null/WT	Retinoblastoma (RB) in Cancer	n/a	2	0.6

Here we describe the fibro-protective effect of TSP1 deficiency in an *in vivo* mouse model of NASH. We initially identified and validated TSP-1 as an anti-fibrotic target of interest by assessing the attenuation of *in vitro* primary human hepatic stellate cell activation by knockdown or pharmacological inhibition (Fig 2). To assess the impact of TSP1 deficiency in context of NASH, we chose CDAHFD model in mice. We observed CDAHFD fed TSP1 knockout mice to have numerous attenuated NASH symptoms, such as decreased serum lipid levels and markers of inflammation (Fig 4). Importantly, we also observed that TSP-1 knockout mice had attenuated hepatic fibrosis by histological measurement (Fig 5). We found that the mRNA level of Collagen I and III, the most abundant type of collagen in fibrotic ECM deposits, not to differ between TSP-1 knockout and WT mice, which may be due to the kinetic difference in mRNA and protein changes in the 12-week study and/or enhanced collagen turnover (Fig 5). A longitudinal study that collects samples for mRNA and protein expression analysis at different time points (e.g. 0, 1, 4, 8, 12 weeks) during the study will be necessary to further characterize the discrepancy found in our study.

Characterization and comparison of transcriptomic profiles of mice by genotype and diet revealed PPARα pathway (Fig 6) and mitochondrial dysfunction, specifically in amino acid metabolism and fatty acid beta oxidation (Fig 7), as pathways and mechanisms that were differentially modulated in TSP-1 knockout mice that may have provided protection against hepatic fibrosis in the CDAHFD model of NASH in mice. Physiological observations such as decreased serum lipid levels and increased serum GLDH levels in TSP-1 knockout mice also supported modulation of these pathways. Our results suggest TSP-1 may be an attractive target in treatment of non-alcoholic steatohepatitis.

We observed a modest attenuation of recombinant TGF-β1 mediated primary human hepatic stellate cell activation *in vitro* by TSP-1 knockdown or by pharmacological inhibition of TSP-1. The modest attenuation may be due to the mechanism by which TSP-1 modulates TGF-β1 mediated HSC activation. TSP-1 activates endogenously released latent TGF- β1 whereas recombinant TGF- β1 does not requires activation. To determine the impact of TSP-1 in TGF- β1 activated HSCs, we utilized recombinant TGF-β1 at EC50 concentration to activate HSC and to initiate the release of endogenous TGF-β1. However, the existence of recombinant active TGF- β1 may have partially masked the magnitude of TSP-1 knockdown/inhibition on the attenuation of activation markers.

TSP-1 has been identified as an important regulator in numerous fibrotic diseases induced by metabolic stress, such as type II diabetes induced diabetic nephropathy and cardiomyopathy. Our study, in reporting TSP-1 deficiency as hepato-protective in a model of non-alcoholic steatohepatitis in mice, further implicates the role of TSP-1 as an important regulator in fibrosis and metabolic dysregulation. Our study observed that WT mice fed CDAHFD exhibit many symptoms of NASH including increase in liver weight, liver enzyme levels, steatosis and fibrosis, consistent with previously reported results of CDAHFD in mice [29]. TSP-1 knockout mice also presented with similar NASH-like symptoms, however with statistically significant attenuation such as decreased serum lipid levels, markers of inflammation (p = 0.02 for Tnf mRNA, p = 0.01 for Tgfb1 mRNA) and fibrosis (p<0.0001, percent area PSR). Serum liver enzyme AST and ALT were not different between TSP-1 knockout and WT mice; however, serum liver enzyme levels do not correlate to histological findings in clinical setting and are unhelpful in diagnosis of NAFLD and in determining the severity of the disease [32]. TSP-1 knockout mice fed CDAHFD also had increased GLDH levels, indicating perturbation in liver mitochondrial dysfunction with TSP-1 deficiency. TSP-1 knockout animals also had significantly lower serum total, LDL and HDL cholesterol level when compared to WT in both control diet and CDAHFD. However, we also observed that CDAHFD fed animals had lower serum lipids than those fed control diet, which seems contraindicative of a high fat diet. This is likely due to choline and methionine deficiency in the diet that impaired the release of VLDL from the liver to induce increased steatosis in the liver. Lastly, there was no significant difference observed between TSP-1 knockout and wild type mice in both diet groups in triglyceride levels suggesting that hepatic fatty acid uptake is not impacted by TSP-1 deficiency.

Our study corroborates with a recent study examining the impact of TSP-1 in a model of obesity. TSP-1 null mice had significantly attenuated mRNA expression of inflammation markers TNF-α and TGF-β1 in adipose tissue from obese mice on high fat diet [23], similar to our observation in the liver. Overexpression of TNF-α in adipose tissues correlates with insulin resistance in animal models, and insulin resistance correlates with onset of NAFLD/NASH. Furthermore, Kong *et al*. also reported attenuated weight gain and serum cholesterol levels in TSP-1 null mice [23], similar to our report. However, Kong *et al*. observed increased triglyceride levels in high fat diet fed TSP-1 null mice whereas in our study did not. This difference is likely due to the impact of choline deficiency and limited methionine in the CDAHFD diet. Similarly, Machado et al. observed decreased triglyceride levels in mice fed choline and methionine deficient diet in comparison to control chow and high-fat Western diet [33]. PCA analysis of our RNAseq data also demonstrated that diet is the greatest driving factor of variance in gene expression, highlighting the overwhelming impact of CDAHFD over genotype in our current study (Fig 5). The purpose for high fat content in CDAHFD is not to drive disease onset and progression; instead, its purpose in mainly to avoid the massive weight loss associated with choline and methionine deficient diet while maintaining the onset of severe liver fibrosis [29] whereas in high fat diet similar to Kong *et al*., high fat content of the diet is the driving factor of obesity onset and progression. Furthermore, mice fed on high fat diet do not develop liver fibrosis, whereas CDAHFD models NASH with robust induction of severe hepatic fibrosis, which may also impact triglyceride uptake in liver and therefore level in circulation.

We observed that TSP-1 KO mice exhibited dramatically decreased hepatic fibrosis *in vivo*; however, we observed modest attenuation of fibrotic markers in TSP-1 knockdown/inhibition in TGF-β1 mediated HSC activation *in vitro*. The difference between *in vivo* and *in vitro* results may be due to additional systemic impact of TSP-1 deficiency in fibrotic pathways, in addition to its role in TGF-β1 activation, such as activation of PPARα pathway. The *in vitro* model system utilizing HSCs did not capture possible cross talks that may exist between different cell types with HSCs, such as hepatocytes and Kupffer cells. Furthremore, surrounding adipose tissues and the gut, can influence and modulate metabolic and inflammatory signals to the liver to influence hepatic fibrosis. Additional experiments to explore the impact of PPARα agonism in TGF-β1 mediated HSC activation *in vitro* and further investigation into cell type- and/or liver- specific TSP-1 KO *in vivo* may elucidate insights into the discrepancy observed between *in vitro* and *in vivo* results.

Our transcriptomic analysis of CDAHFD fed TSP-1 knockout mice also indicated modulation of the amino acid metabolism pathway. Liver is the site of synthesis and catabolism of protein and amino acid where the mitochondria play a key role via the tricarboxylic acid (TCA) cycle, ATP synthesis and mitochondrial respiration [34]. Increased amino acid concentrations have been associated with increased risk of metabolic diseases such as type 2 diabetes and NAFLD [35]. Plasma free amino acid profiles have also been correlated with progression of fatty liver diseases with NASH patients exhibiting highest values [36]. Significant decrease in genes associated with branched chain amino acid catabolism and fatty acid beta oxidation has been observed in NAFLD patients [37] which correlates with our data comparing CDAHFD fed mice with control diet fed mice that also showed downregulation of fatty acid beta oxidation and amino acid metabolism. Therefore, dysregulated amino acid metabolism is associated with development of NAFLD/NASH. However, the exact mechanism behind how dysregulated amino acid metabolism contributes to NAFLD is not well defined.

Interestingly, Soto-Pantoja *et al*. also recently reported differentially regulated elements of mitochondrial dysfunction in TSP-1 knockout mice. Amino acid and lipid metabolism as well as and ketone body formation were identified by global metabolic profiling study that examined the impact of TSP-1 deficiency combined with high fat diet [38]. Specifically, in TSP-1 deficient liver, ketogenic amino acid and lipid metabolism changes associated with dysregulated TCA cycle were observed, suggesting mitochondrial dysfunction in TSP-1 knockout mice [38]. Similarly, we also observed an increase in serum glutamate dehydrogenase (GLDH) in TSP-1 knockout animals fed CDAHFD. GLDH is an enzyme important in amino acid oxidation and urea production pathway. [39]. McGill *et al*. recently proposed GLDH as a biomarker of liver mitochondrial dysfunction as increased GLDH serum concentration was observed in mice with hepatic injuries [40, 41]. Our study indicated an increase in GLDH in TSP-1 knockout mice fed CDAHFD, also suggesting a possible increased mitochondrial dysfunction with TSP-1 deficiency. However, our transcriptomic data suggests upregulation of amino acid metabolism and fatty acid beta oxidation in TSP-1 deficient mice which suggests improved mitochondrial function. A key difference to note between our study and Soto-Pantoja *et al*. is that their study examined impact of high fat diet on TSP-1 deficiency in Apc ^*min/+*^ mice, whereas our study examined the impact of choline deficient, limited methionine and high fat diet driven model of hepatic fibrosis with TSP-1 deficiency in C57BL/6J background. The differences in genotypes and disease model may lead to different activation of various pathways modulated by TSP-1. Because mitochondrial dysfunction may be manifested in numerous ways, it is also possible that TSP-1 deficiency may improve one facet such as amino acid metabolism while worsening another such as ketogenesis. Based on our data and the data from Soto-Pantoja *et al*., TSP-1 modulation clearly impacts mitochondrial function in the context of metabolic stress. However, further investigations are necessary to elucidate mechanisms and physiological meaning underlying the relationship between TSP-1, amino acid metabolism, and mitochondrial dysfunction in NAFLD.

Our study suggests that TSP-1 plays an important role in NAFLD/NASH by modulation of PPARα pathway. PPARα has recently been identified as a target of interest and a new potential therapeutic area for NAFLD/NASH. Activation of PPARα in combination with PPARβ/δ improves steatosis, inflammation and fibrosis in a preclinical model of non-alcoholic fatty liver disease [42]. Fibrates are a class of PPAR agonists that are approved for treating various dyslipidemias such as hypercholesterolemia by reducing very low density lipoproteins (VLDL) production in the liver. Fenofibrate treatment of patients with NAFLD resulted in reduction of elevated ALT, AST and γGT plasma levels and hepatocellular ballooning in some patients; however, steatosis, inflammation and fibrosis were not significantly changed [43]. Treatment of NASH patients with another fibrate, gemfibrozil, also lowered ALT, AST and γGT [44]; however, in contrast, clofibrate, another PPAR agonist, did not improve ALT, AST, γGT or histological assessment of steatosis inflammation and fibrosis [45]. Another PPARα and PPARγ agonist compound, pioglitazone, was found to improve NAFLD in diabetic and non-diabetic patients [46, 47]. The differences observed in outcomes with various PPAR agonists may be due to differences in their selectivity for PPAR receptor subtypes. Further investigations into the mechanism of PPARα activation, possibly via TSP-1 modulation, in NASH-induced liver fibrosis may be beneficial in the development of novel therapies for NASH.

We also observed modulation of mitochondrial function in TSP-1 knockout mice. Mitochondrial dysfunction as a target has also been recently explored as a treatment strategy for NASH. Mitochondrial oxidative metabolism and hepatic inflammation were observed to be closely linked [48]. Furthermore, targeting mitochondrial function in animal studies has shown reduction in hepatic steatosis, necrosis, inflammation and fibrotic progression in NASH [48, 49]. However, whether these studies are directly translatable to human disease has not yet been explored.

## Conclusions

In summary, our study demonstrates that TSP-1 plays a role in NAFLD/NASH and modulates PPARα pathway and amino acid metabolism *in vivo*, as well as hepatic stellate cell activation *in vitro*. TSP-1 deficiency attenuated hepatic fibrosis and other NAFLD symptoms such as serum lipid levels and inflammation markers. Furthermore, TSP-1 deficiency mediated modulation of PPARα pathway in CDAHFD induced hepatic fibrosis were observed in both physiological and transcriptomic data. Although amino acid metabolism was identified in transcriptomic analysis, the exact role of its modulation and impact on NAFLD/fibrosis must be investigated further. In combination with corroborative findings by other studies examining the role of TSP-1 and metabolic dysfunction mediated fibrosis [18, 19, 23–25], our study suggests that TSP-1 may be an attractive target for NASH-induced hepatic fibrosis.

## Supporting information

S1 Fig Raw images of Fig 2A and 2B. (PDF)
