# Supplementary material for: Thrombospondin-I is a critical modulator in non-alcoholic steatohepatitis (NASH)
Source: PLoS One. 2019 Dec 31;14(12):e0226854. doi: 10.1371/journal.pone.0226854 (PMC6938381; doi:10.1371/journal.pone.0226854)
Supplement: S1 Fig — (PDF) [file pone.0226854.s001.pdf]

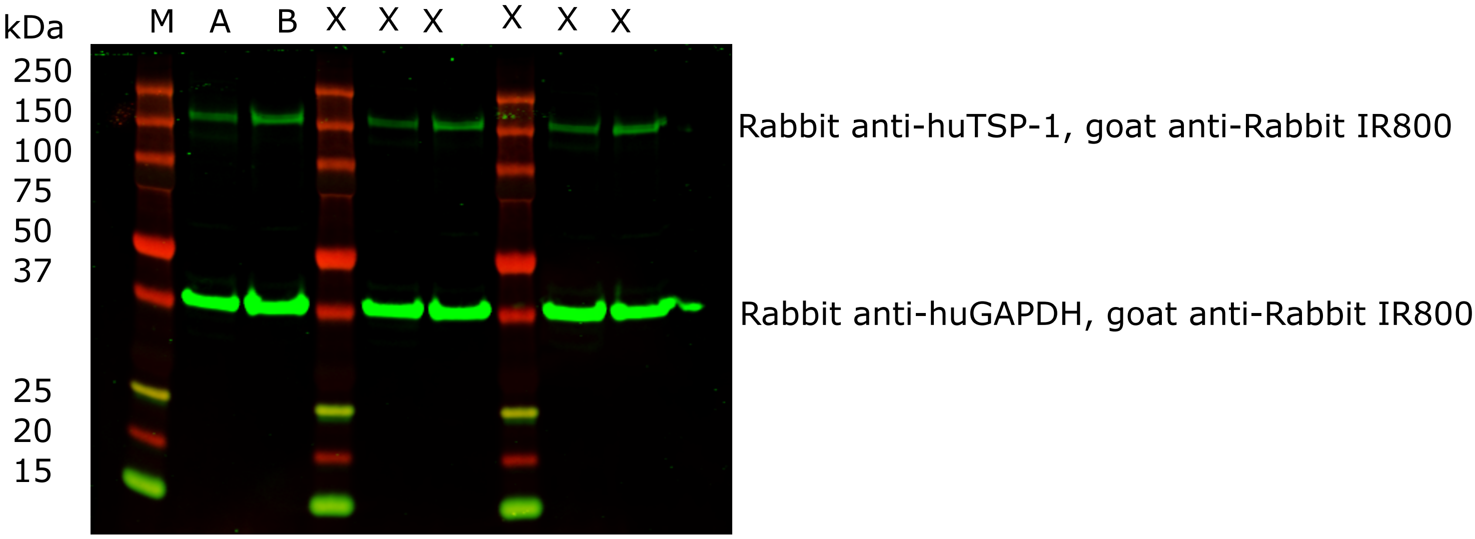

Raw western blot image of figure 2A.
Lane M is Biorad Precision Plus Protein Dual color standard
Lane A is vehicle treated human hepatic stellate cells
Lane B is human hepatic stellate cells treated with TGFb1
Image was acquired using Li-Cor Odyssey CLx system and Image Studio software.
Both 800 andd 700 channel laser source was used to visualize the bands (green and red).

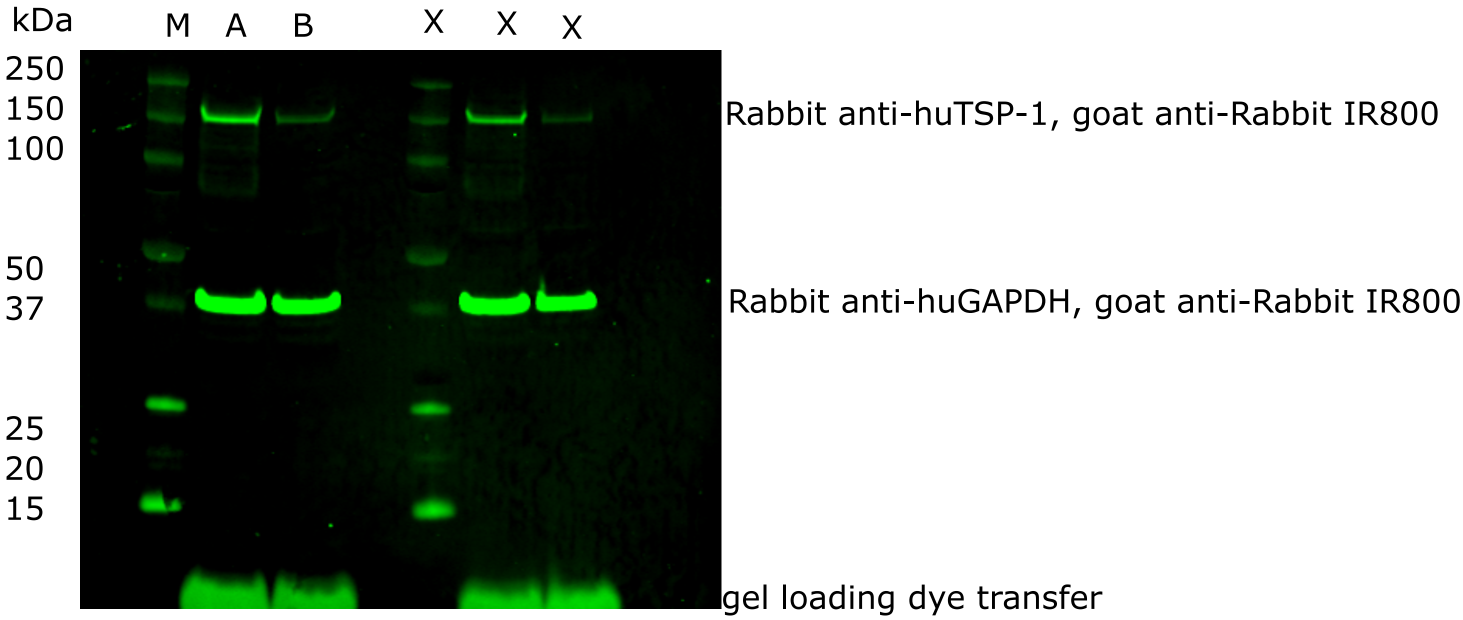

Raw western blot image of figure 2B.
Lane M is Biorad Precision Plus Protein Dual color standard
Lane A is TGFb1 activated human hepatic stellate cells transduced with Adeno-control shRNA
Lane B is TGFb1 activated human hepatic stellate cells transduced with Adeno-shTSP-1
Image was acquired using Li-Cor Odyssey CLx system and Image Studio software.
Channel 800 laser source was used to visualize the bands(green).
